# 322. Improving Assessment of Beta-Lactam Allergies by Embedding a Questionnaire in Best Practice Advisory Alerts Across a Large Health System

**DOI:** 10.1093/ofid/ofad500.393

**Published:** 2023-11-27

**Authors:** Michael S Boger, Michael S Boger, Rupal K Jaffa, Rebecca Kiliany, Ekta Shah, Lee Morris, Lisa Davidson

**Affiliations:** Atrium Health, Charlotte, North Carolina; Atrium Health, Charlotte, North Carolina; Atrium Health, Charlotte, North Carolina; Atrium Health, Charlotte, North Carolina; Atrium Health, Charlotte, North Carolina; Atrium Health, Charlotte, North Carolina; Atrium Health, Charlotte, North Carolina

## Abstract

**Background:**

Penicillin (PCN) allergy is commonly reported. Yet most patients labeled allergic can tolerate beta-lactams (BLs), the antibiotics of choice for many infections. Documentation of reaction details is important to identify low risk patients who can safely receive BLs, but it is often incomplete. A May 2018 point prevalence study from the electronic medical record (EMR) using a one-week sample of Atrium Health Greater Charlotte Market (AH) PCN allergy patients showed that 52% had no reaction documented.

**Methods:**

This was a retrospective, observational study in thirteen hospitals in the AH system. To improve BL allergy assessment, a BL allergy questionnaire was developed during the launch of a new AH wide EMR. During the initial roll-out, a best practice advisory (BPA) fired for nursing when entering a BL allergy, prompting a link to a separate BL allergy questionnaire for nursing completion. After all sites were live, we assessed questionnaire completion rates. During the optimization phase, the questionnaire was incorporated directly in the BPA and simplified from ten to three questions, allowing completion in one step (Fig 1). The answers are then automatically sent to flowsheets where they are available for reference and feed into assessment algorithms that provide the allergy level of risk and clinician treatment guidance upon antibiotic order entry (Fig 2). The BPA fires daily as a reminder until questionnaire completion.

**Figure 1.**
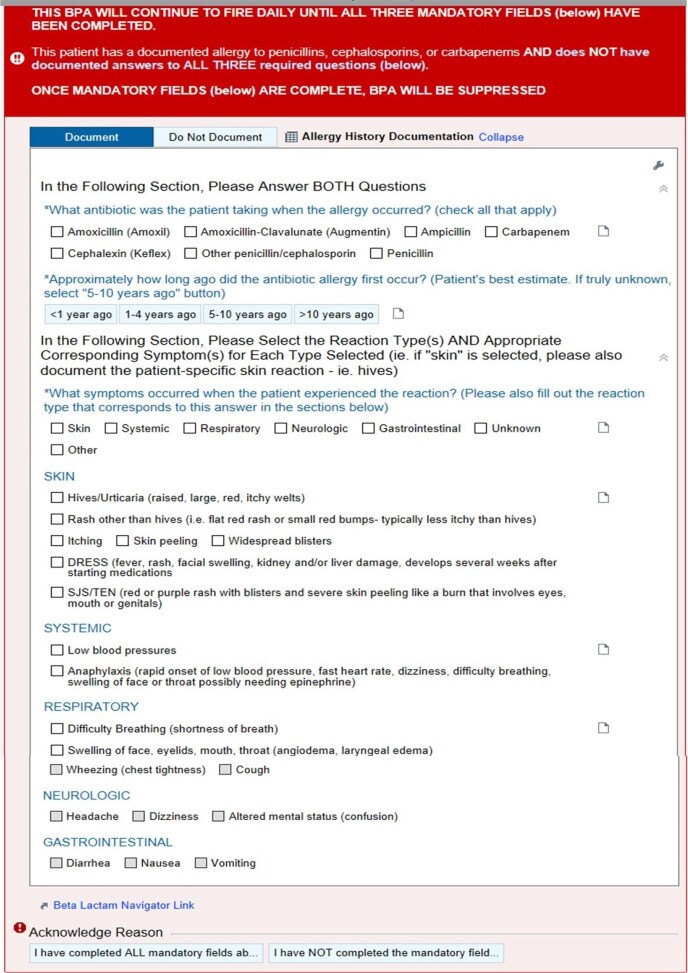
Beta-Lactam Allergy Questionnaire Best Practice Advisory (BPA).

**Figure 2.**
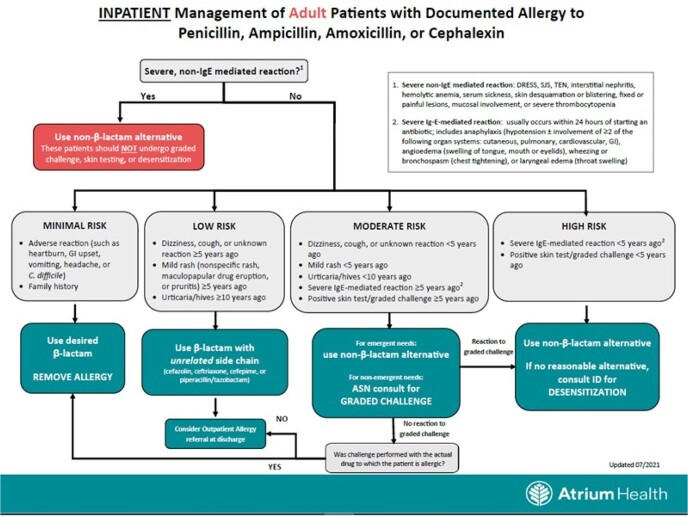
Beta-Lactam Allergy Assessment Algorithm.

**Results:**

**Pre-optimization:** Over a ten month period from December 2021 through October 2022, 4118 inpatient questionnaires were completed. Beginning in August 2022, when all facilities were live on the new EMR, the average monthly questionnaire completion was 833 (Fig 3). **Post-optimization:** In November 2022, completion increased 38.9%, resulting in an additional 4743 questionnaires over four months (average of 1186 per month). The number of questionnaires completed thereafter decreased as the number of patients with previously completed questionnaires increased.

**Figure:**
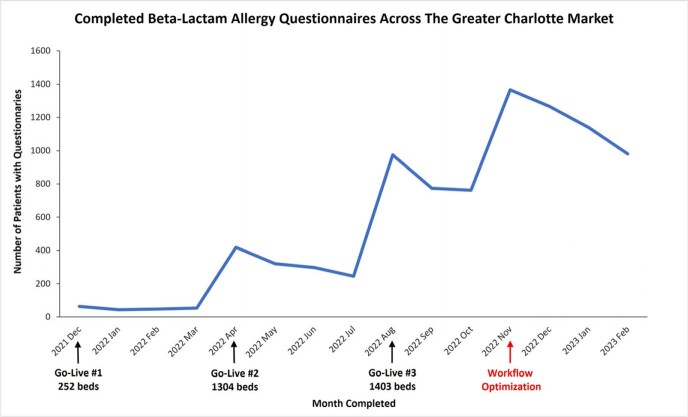

**Conclusion:**

The creation of a targeted BL allergy questionnaire in the EMR improved BL allergy documentation across a large, diverse health system. The effect was enhanced through workflow optimization incorporating the questionnaire into a one-step BPA.

**Disclosures:**

**All Authors**: No reported disclosures

